# Treatment of Fibroblast Growth Factor-23-Mediated Renal Phosphate Wasting With Burosumab in a Patient With Chronic Kidney Disease 3b: A Case Report

**DOI:** 10.7759/cureus.99956

**Published:** 2025-12-23

**Authors:** Christopher H Goss, Geethika Earthineni, Vishwajeeth Pasham, Tibor Fülöp

**Affiliations:** 1 Nephrology, Medical University of South Carolina, Charleston, USA; 2 Pathology and Laboratory Medicine, Medical University of South Carolina, Charleston, USA; 3 Medical Services, Ralph H. Johnson VA Medical Center, Charleston, USA

**Keywords:** burosumab, calcium-phosphorus imbalance, chronic kidney disease (ckd), electrolytes abnormalities, elevated fgf23, endocrinology and diabetes, general internal medicine, general nephrology, symptomatic hypophosphatemia

## Abstract

We describe the case of a 72-year-old man with chronic kidney disease (CKD) 3b due to hypertensive nephrosclerosis, noted to have persistent hypophosphatemia and mildly elevated fibroblast growth factor-23 (FGF-23). His serum phosphorus level was chronically low, averaging 1.6 mg/dL, despite taking six to eight 250 mg packets of an oral elemental phosphate supplement daily. His parathyroid hormone (PTH) level was 204 pg/mL (normal range: 10-65 pg/mL), and his serum alkaline phosphatase (ALP) level was within the normal range at 55 U/L. His fractional excretion of phosphate was found to be 59%, confirming excessive urinary losses as the primary reason for the low serum phosphorus levels. Numerous PET/CT scans failed to localize an FGF-23-producing tumor. As part of his treatment plan, he was started on burosumab 50 mg injected subcutaneously every four weeks. Within six weeks, his serum phosphorus normalized, and oral supplementation was discontinued. His PTH level normalized, and his kidney function stabilized after an acute kidney injury. No further adverse events occurred, and he tolerated the burosumab without issue. This current case illustrates successful correction of renal phosphate wasting with burosumab in a patient with CKD and non-localizable FGF-23. This suggests that targeted anti-FGF-23 therapy can be considered for intolerable or ineffective conventional therapy even in moderate CKD.

## Introduction

Fibroblast growth factor-23 (FGF-23) is a phosphaturic hormone produced by osteocytes, and excessive activity leads to renal phosphate wasting and hypophosphatemia [1]. An excess secretion of FGF-23 causes hypophosphatemia and is classically described as tumor-induced osteomalacia (TIO), a condition with FGF-23-producing tumors [1,2]. Typically, these tumors are small, and when no tumor can be found, definitive treatment is challenging. Treatment in this case typically focuses on oral phosphate supplementation, but this treatment rarely normalizes serum phosphorus levels alone. Burosumab, a monoclonal antibody targeted against FGF-23 and approved for TIO and X-linked hypophosphatemia (XLH), has not been extensively studied in patients with chronic kidney disease (CKD) and elevated FGF-23 levels.

Phosphate balance is maintained by intestinal absorption, bone turnover, and renal excretion. The FGF-23 decreases phosphate reabsorption in the proximal convoluted tubule (PCT) and inhibits renal 1-α-hydroxylase. Inhibition of 1 α-hydroxylase suppresses calcitriol (activated vitamin D) synthesis. In patients with chronic overproduction of FGF-23, renal phosphate wasting and hypophosphatemia are characteristic. Symptomatic phosphate wasting can be characterized by bone pain, fractures, weakness, and myopathy. The classic acquired cause can include TIO, in which a small tumor secretes FGF-23 [1,2].

When found on imaging, this condition can essentially be cured with surgical resection of the tumor. In patients who have elevated FGF-23 but no identifiable tumor, these patients typically take high-dose oral phosphate and activated vitamin D analogs to maintain serum phosphate. However, aggressive oral phosphate supplementation can lead to gastrointestinal intolerance, nephrocalcinosis, secondary hyperparathyroidism, and worsening of renal function due to phosphate-associated acid load [3,4].

Burosumab is a monoclonal antibody targeted against FGF-23. By inhibiting FGF-23, Burosumab increases tubular phosphate reabsorption and serum calcitriol. It was first approved for XLH in 2018 and then later for TIO, but only when a tumor cannot be localized or resected [5,6]. Burosumab has not been extensively studied in CKD populations [7]. The clinical use of burosumab in CKD patients with proven renal phosphate wasting remains poorly documented, and this case report aims to address this gap.

## Case presentation

A 72-year-old man with CKD 3b due to hypertensive nephrosclerosis presented to the office for evaluation. His past medical history included a surgically resected pituitary adenoma complicated by subsequent panhypopituitarism on hormone therapy, atrial fibrillation on apixaban, numerous renal cysts, hypothyroidism on levothyroxine, hypertension, hypertrophic cardiomyopathy, obstructive sleep apnea, gout, benign prostatic hyperplasia, and osteoarthritis. He had been noted to have low serum phosphate for over a decade, generally ranging between 1.5 and 2.5 mg/dL despite aggressive supplementation with oral potassium phosphate. He reported chronic fatigue and muscle weakness, but there was no history of fractures. His muscle weakness was predominantly proximal and exertional, without gait instability or falls. Notably, he had no history of gastric surgery and no history of malabsorptive intestinal disease.

Laboratory values showed serum phosphorus of 1.6 mg/dL, calcium 9 mg/dL, creatinine 1.57 mg/dL, bicarbonate 24 mEq/L, and parathyroid hormone (PTH) 204 pg/mL. A 24-hour urine collection revealed volume 2075 ml, phosphorus 1,600 mg (normal 400-1300 mg/24 hrs), and creatinine 1.84 g. The fractional excretion of phosphate was 59%. Serum FGF-23 measured in 2016 was 76 pg/mL (normal < 6.5 pg/mL), and 185 RU/mL (normal < 180) in 2022. The 1,25-dihydroxy vitamin D was normal at 41 pg/mL. A summary of key laboratory findings is shown in Table 1. The patient’s chronic baseline phosphorus of 1.5-2.5 mg/dL represents longstanding disease and does not modify laboratory interpretation; all low/high (L/H) categorizations reflect the actual lab reference range.

**Table 1:** Longitudinal laboratory data before and after burosumab initiation PTH: Parathyroid hormone; ALP: Alkaline phosphatase; eGFR: Estimated glomerular filtration rate; SC: Subcutaneous; Ref: Reference range; L: Low relative to reference range, H: High relative to reference range; N/A: Not available. The L/H categorization refers strictly to laboratory reference intervals.

Date	Clinical phase	Phosphorus mg/dL (ref: 2.5-4.9)	Calcium mg/dL (ef: 8.5-10.2)	PTH pg/mL (ref: 15-65)	ALP U/L (ref: 44-147)	Creatinine mg/dL (ref: 0.6-1.3)	eGFR mL/min/1.73 m²	Magnesium mg/dL (ref: 1.7-2.4)
5/27/2022	Pre-treatment baseline	2.2 L	8.7	N/A	56	1.73 H	40	1.68 L
5/17/2023	Pre-burosumab (baseline)	1.6 L	9	204 H	55	1.57 H	46	N/A
6/12/2023	Burosumab initiation (50 mg SC)	1.7 L	8.4 L	N/A	55	1.51 H	49	1.66 L
7/5/2023	Three weeks post-dose	4.2	9.3	N/A	47	1.63 H	40	1.66 L
7/24/2023	Six weeks post-dose	2.8	9.4	N/A	46	1.85 H	31	1.47 L
8/28/2023	10 weeks post-dose	3.1	9.1	N/A	44 L	1.89 H	34	1.79
9/19/2023	Peak creatinine	3.4	8.5	N/A	48	2.19 H	31	N/A
10/30/2023	Renal biopsy	3.3	8.6	N/A	49	1.95 H	36	1.63 L
11/17/2023	Early recovery	3.7	8.7	N/A	45	1.92 H	37	1.77
12/15/2023	Six months post-dose	4.5	9	87 H	45	1.94 H	36	N/A
3/5/2024	Nine months post-dose	3.5	9	N/A	47	1.92 H	39	N/A
5/8/2024	11 months post-dose	2.1 L	8.8	N/A	43	2.00 H	34	N/A
7/24/2024	13 months post-dose	2.8	9.4	N/A	46	2.16 H	31	1.66 L
9/30/2024	15 months post-dose	3	9.7	N/A	43 L	1.79 H	34	1.79
12/3/2024	18 months post-dose	2.4 L	9	N/A	50	1.78 H	40	N/A
3/5/2025	21 months post-dose	3.5	9.4	N/A	47	1.84 H	39	1.84
7/24/2025	25 months post-dose	2.8	9.4	N/A	46	1.83 H	40	1.66 L

The PET/CT in 2016 showed a lesion on the patient’s right tonsil. He underwent ENT evaluation; the biopsy of the lesion was negative for malignancy. On subsequent imaging, there was no abnormal uptake in the tonsil lesion. Based on these findings and labs, the nephrology and endocrinology team diagnosed an FGF-23-mediated disorder without an identified tumor.

The patient had difficulty with the oral phosphate supplementation and notably had side effects, including dose-limiting gastrointestinal intolerance, with worsening diarrhea above six packets daily, as well as acidemia noted on labs. Because of these side effects, a trial of burosumab was approved. The rationale included the potential renal toxicity of chronic inorganic phosphate and acid load and the failure of conventional supplementation to normalize his phosphate levels. Within one month of starting burosumab, his serum phosphorus increased from 1.6 mg/dL to 4.1 mg/dL. His phosphorus stabilized in the low-normal range between 2.6 and 4.1 mg/dL approximately one month later, without supplementation (Table 1).

**Figure 1:**
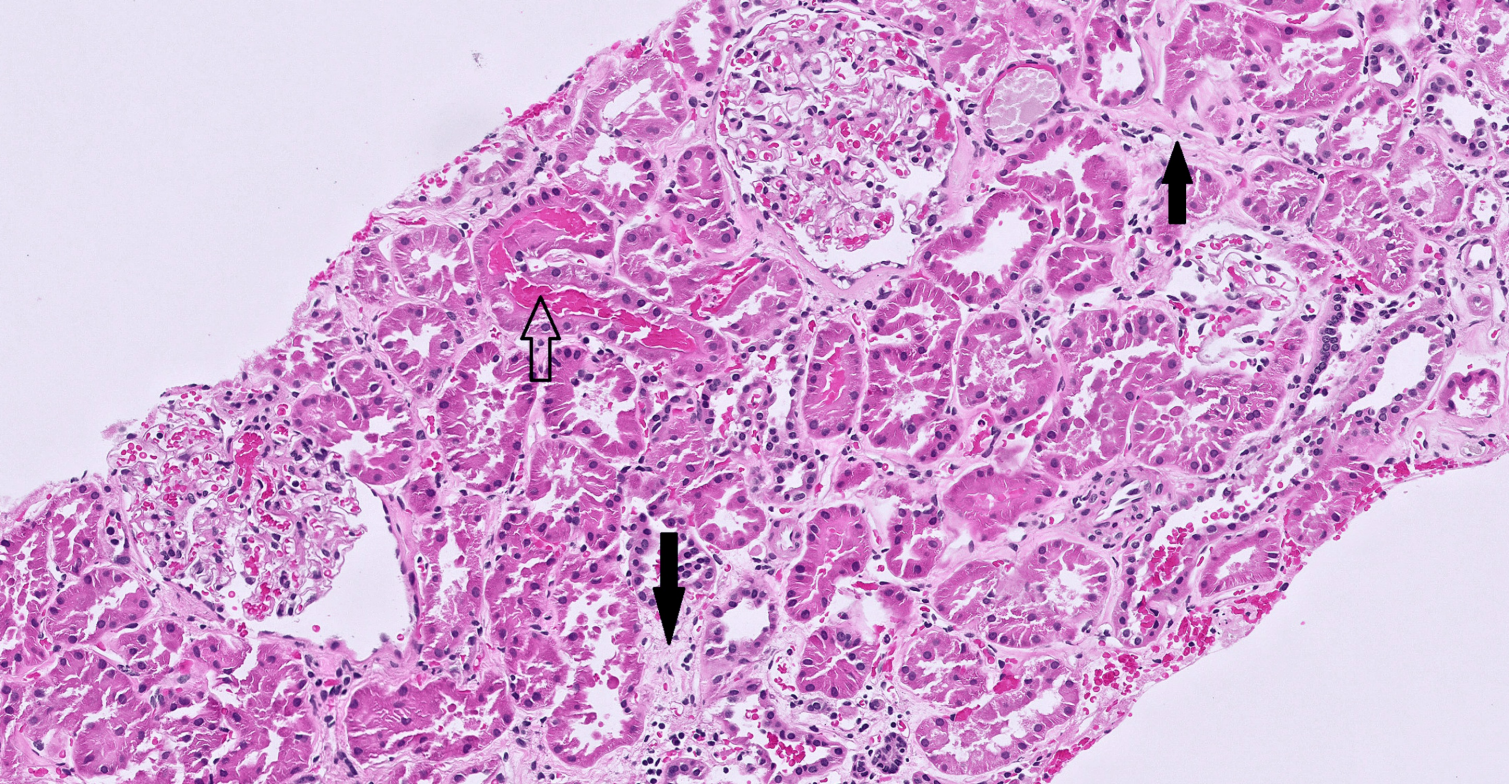
Renal biopsy demonstrating chronic changes consistent with hypertensive nephrosclerosis (hematoxylin and eosin stain, 200×) There is mild to moderate interstitial fibrosis and tubular atrophy (black arrows), no evidence of proliferative glomerulonephritis, allergic interstitial nephritis, acute tubular injury, or crystal nephropathy. A few fresh red blood cells are seen within tubules (arrow outline), which can be due to bleeding during the biopsy rather than representing red blood cell casts.

To further complicate the interpretation of this scenario, he also experienced a mild, transient rise in serum creatinine after initiation of burosumab, peaking at 2.1 mg/dL from a baseline of 1.6 mg/dL, while serum phosphorus normalized. Renal biopsy at that time revealed moderate chronic changes with arteriosclerosis and arteriolar medial hypertrophy, consistent with hypertensive nephrosclerosis, without evidence of immune complex disease, phosphate deposition, or tubular injury (Figure 1). The biopsy findings supported that the kidney function change represented hemodynamic fluctuation rather than drug-related nephrotoxicity; there were no biopsy findings to suggest tubular injury or mineral deposition attributable to burosumab. His renal function eventually returned to baseline; urea and creatinine clearances averaged an eGFR of 49 mL/min, consistent with stable CKD 3 a-b transition.

Over the subsequent months, the patient reported increased exercise tolerance. The patient had no muscle cramping or gout flares. His renal function remained stable. The PTH normalized to 87 pg/mL, hemoglobin remained stable, and magnesium deficiency was mild. On his most recent clinic visit, the patient continued burosumab injections every four weeks. Serum phosphorus remained within normal range without supplementation, and renal function was unchanged. He was maintained on metoprolol 12.5 mg BID, sodium bicarbonate 650 mg BID, and magnesium oxide 400 mg daily. He described good physical activity and no adverse effects.

An earlier FGF-23 value from 2016 (reported as 76 pg/mL) was determined to be erroneous due to assay mislabeling; subsequent reliable measurements were 185 RU/mL in 2022 and 178 RU/mL in 2023 (normal <180 RU/mL). The patient’s 1,25-dihydroxyvitamin D and calcium levels remained normal, arguing against secondary hyperparathyroidism or vitamin D-deficient bone disease as primary causes.

Burosumab therapy was continued with monthly laboratory monitoring. Phosphate values remained in the low-normal range, with return of renal function to baseline without new adverse effects. This sequence supports the conclusion that FGF-23-driven phosphate wasting persisted despite modest absolute FGF-23 elevation and that targeted antibody therapy provided physiologic correction without progressive renal compromise.

## Discussion

This case shows that burosumab can effectively correct FGF-23-mediated phosphate wasting safely, even in the presence of CKD. The patient had biochemical features typical of FGF-23 excess, and although his FGF-23 elevation was modest compared to the extreme levels seen in classic TIO, the overall physiology was consistent with excessive phosphaturia.

In many patients with TIO, localization of the responsible tumor is often not feasible. As many as 35% to 40% of cases fail to yield a detectable lesion despite imaging [8]. In cases where imaging is negative, long-term oral phosphate and calcitriol have been used, but such regimens are difficult to maintain and can have negative effects on renal function. Chronic phosphate supplementation imposes an acid load that may promote tubulointerstitial injury and accelerate CKD progression [4,8].

Burosumab works by binding to circulating FGF-23 and preventing its interaction with renal FGFR-Klotho complexes. This restores sodium-phosphate co-transporter expression in the PCT [9]. In trials of adult patients with TIO, normalization of serum phosphate and fracture healing over 144 weeks was noted [7]. Adverse effects, when described, were generally mild and included local injection-site reactions and transient elevations in phosphate and creatinine.

Most studies on burosumab excluded participants with advanced CKD, leaving uncertainty about safety in reduced renal function. This case provides further evidence that moderate CKD does not preclude efficacy and safety in using burosumab. The patient has demonstrated stability of renal function over more than 18 months (after initial acute kidney injury (AKI)) without development of hyperphosphatemia or renal calcium stones. Normalization of phosphate balance occurred promptly, along with a decrease in PTH to within the target range, suggesting an overall improvement in bone-mineral metabolism.

The response also underscores that FGF-23 can be pathogenic even when elevations are modest. In CKD, serum FGF-23 levels rise as a compensatory mechanism to increase phosphate excretion. Regardless of the etiology, the pathophysiologic mechanism was directly targeted and corrected [1-4]. Any interpretation of elevated FGF-23 should be tempered by the knowledge of elevated FGF-23 levels in CKD patients, and formal establishment of excess and inappropriate wasting of phosphorus is a priority.

The XLH was unlikely in our index case, given the late presentation of the patient’s symptoms and the lack of clinical symptoms, such as osteomalacia, bone fractures, or overt dental disease. To clarify, XLH is caused by a loss-of-function mutation in the phosphate-regulating endopeptidase homolog X-linked (PHEX) gene, which normally regulates FGF-23 production, leading to uncontrolled FGF-23 production and hypophosphatemia [10,11].

Due to concern about hyperphosphatemia and extra-skeletal calcification, use of burosumab is not indicated in severe renal disease [5,10]. In moderate CKD, risk can be diminished with careful laboratory monitoring. Our patient underwent monthly phosphorus and renal function panels and annual imaging, without evidence of mineral deposition. Limited evidence in sporadic case reports suggests that the cautious use of burosumab in selected patients with CKD may be appropriate [11]. Our case adds to these reported scenarios, explores the use of burosumab in adult-onset disease, and also highlights that modest FGF-23 elevations in CKD may still exert physiologically inappropriate phosphaturic effects when urinary losses remain excessive despite normal or low serum phosphate.

## Conclusions

The report suggests that for a CKD patient with elevated FGF‑23, a targeted antibody regimen might be an appropriate alternative when conventional treatment is less appealing. Vigilant long‑term monitoring remains essential to catch late‑emerging complications, evaluate bone health, and decide whether therapy must be continued indefinitely. In this case, burosumab restored phosphate balance and improved the biochemical profile of a CKD 3b patient with FGF‑23‑driven renal phosphate wasting. The patient tolerated the therapy without difficulty. This case hints that blocking FGF‑23 can be both safe and effective, provided the treatment strategy is grounded in an understanding of the underlying pathophysiology and is accompanied by close monitoring. The conclusions from a single carefully monitored patient must be interpreted cautiously, and broader generalizability cannot be assumed. Important evidence gaps remain regarding long-term renal safety, criteria for patient selection, and whether dosing can eventually be escalated, tapered, or discontinued. Because TIO-analog phenotypes are uncommon, a multicenter prospective registry of CKD patients with confirmed FGF-23-mediated phosphate wasting treated with burosumab could be a feasible next step to clarify safety signals, patient response, and real-world treatment patterns.
